# Sequence Type Changes Associated with Decreasing Macrolide-Resistant *Mycoplasma pneumoniae*, Japan

**DOI:** 10.3201/eid2609.191575

**Published:** 2020-09

**Authors:** Miyuki Morozumi, Takeshi Tajima, Megumi Sakuma, Michi Shouji, Hidenori Meguro, Kota Saito, Satoshi Iwata, Kimiko Ubukata

**Affiliations:** Keio University School of Medicine, Tokyo, Japan (M. Morozumi, M. Sakuma, S. Iwata, K. Ubukata);; Hakujikai Memorial Hospital, Tokyo (T. Tajima);; National Center for Global Health and Medicine, Tokyo (M. Shouji);; Meguro Clinic, Chiba, Japan (H. Meguro);; Saito Pediatric Clinic, Saitama, Japan (K. Saito);; National Cancer Center Hospital, Tokyo (S. Iwata)

**Keywords:** Mycoplasma pneumoniae, bacteria, macrolide resistance, antimicrobial resistance, multilocus sequence typing, sequence type, Japan

## Abstract

We compared sequence types (STs) of *Mycoplasma pneumoniae* isolates from Japan during 2002–2019. ST3 and ST14 dominated during 2002–2016, and ST7 and ST33 dominated during 2018–2019. These STs were associated with a decrease in macrolide-resistant strains after an epidemic of infection with *M. pneumoniae* during 2011–2012.

*Mycoplasma pneumoniae* is a major cause of community-acquired pneumonia, and macrolide-resistant *M. pneumoniae* is a serious concern in Asia (*1**–**3*). Throughout Japan, an outbreak of macrolide-resistant *M. pneumoniae* infection occurred during 2011–2012 (*2*). After this outbreak, the number of drug-resistant strains decreased for every year from 2013 through 2019. In contrast, China and South Korea still showed a high rate of macrolide resistance in *M. pneumoniae* during 2014–2018 (*1*,*3*). We determined antimicrobial drug susceptibility and performed analysis by using multilocus sequence typing (MLST), clonal complexes (CCs), and P1 gene typing for *M. pneumoniae* isolated from children to identify trends concerning this bacterium in Japan.

## The Study

We obtained nasopharyngeal swab samples from patients who had pneumonia or bronchitis at 21 medical institutions throughout Japan during October 2018–July 2019. We collected samples after obtaining informed consent from patients or their family members (Ethics Committee approval no. 2016–0015, Keio University School of Medicine, Tokyo, Japan).

We suspended samples in 0.5 mL of pleuropneumonia-like organism broth (Difco, https://www.fishersci.com). We then performed DNA extraction by using a described protocol (*4*). We used a Cycleave PCR Kit (Takara Bio, https://www.takarabio.com) to detect *M. pneumoniae*. For confirmed cases of infection with *M. pneumoniae*, we used a Cycleave PCR to distinguish between macrolide-susceptible and macrolide-resistant strains (*5*). Cultures were grown in pleuropneumonia-like organism broth, according to previously described methods (*6*).

We determined MICs for antimicrobial resistance of isolates by using microdilution methods (*6*). We performed MLST analysis based upon sequencing of 8 housekeeping genes (*ppa*, *pgm*, *gyrB*, *gmk*, *glyA*, *atpA*, *arcC*, and *adk*) according to the method described in the MLST database (https://pubmlst.org/mpneumoniae). To determine relationships between sequence types (STs), we performed CC analysis by using global optimal eBURST (http://www.phyloviz.net/goeburst). Typing of the P1 adhesin gene in *M. pneumoniae* was performed as described (*7*).

During the 2018–2019 study period, 105 samples were received (mean patient age 8 years). *M. pneumoniae* was confirmed by real-time PCR in 83 (79.0%), and culturing was successful in 53 (50.5%). Of these 53 isolates, only 6 (11.3%) were macrolide-resistant *M. pneumoniae*. All of these macrolide-resistant strains had an A2063G mutation in the 23S rRNA gene.

We provide yearly changes in macrolide-resistant *M. pneumoniae* during 2002–2019 (except for 2014 and 2017) in Japan (Table). Data from the earlier years beginning in 2002 were reported previously (*2*,*6*,*7*). Our study group results from the earlier periods indicated macrolide-resistance rates of 6.9% (18/259) during 2002–2005; a total of 37.4% (96/257) during 2006–2009; a total of 86.2% (281/326) during 2010–2013, including the epidemic years 2011–2012; and 56.3% (111/197) during 2015–2016 compared with 11.3% (6/53) during 2018–2019. These resistance rates have decreased rapidly beginning in 2018, and the MICs for quinolone and tetracycline have remained unchanged; no drug-resistant strains were identified.

**Table:** Yearly changes in macrolide-susceptible and macrolide-resistant *Mycoplasma pneumoniae*, Japan, 2002–2019*

Macrolide susceptibility†	2002–2005	2006–2009	2010–2013	2015–2016	2018–2019	Total
Susceptible	241 (93.1)	161 (62.6)	45 (13.8)	86 (43.7)	47 (88.7)	580
Resistant	18 (6.9)	96 (37.4)	281 (86.2)	111 (56.3)	6 (11.3)	512
Total	259	257	326	197	53	1,092

*Values are no. (%). †Macrolide susceptibility was distinguished on the basis of a mutation in the 23S rRNA gene and results of susceptibility measurements.

**Figure 1:**
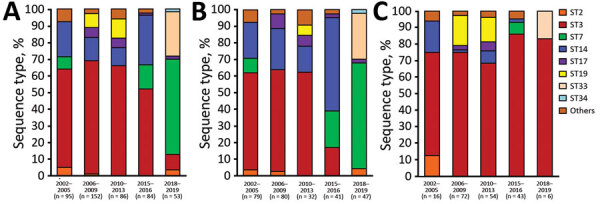
Relationships between year of isolation and STs among 279 macrolide-susceptible *M. pneumoniae* and 191 macrolide-resistant *M. pneumoniae* from children in Japan, 2002–2019. A) All strains tested; B) macrolide-susceptible strains; C) macrolide-resistant strains. Others includes ST13 (2005), ST15 (2002, 2016), ST16 (2002, 2010), ST18 (2010), ST20 (2004), ST21 (2011), ST 22 (2003, 2006, 2016), ST29 (2016), and ST30 (2016). ST, sequence type.

We determined relationships observed between STs and 279 macrolide-susceptible *M. pneumoniae* versus 191 macrolide-resistant *M. pneumoniae* during 2002–2019 (Figure 1). ST3 and ST14 accounted for most macrolide-susceptible *M. pneumoniae* during 2002–2016; these STs have been largely replaced by ST7 (n = 30, 56.6%) and ST33 (n = 13, 24.5%) during 2018–2019. ST33 first appeared in this study in 2018, and ST14 was more prevalent among macrolide-susceptible *M. pneumoniae* during 2002–2016 but was rarely detected during 2018–2019. Differences in STs during 2002–2016 and during 2018–2019 were highly significant (p<0.001). Conversely, most macrolide-resistant *M. pneumoniae* isolates belonged to ST3, as in previous years.

**Figure 2:**
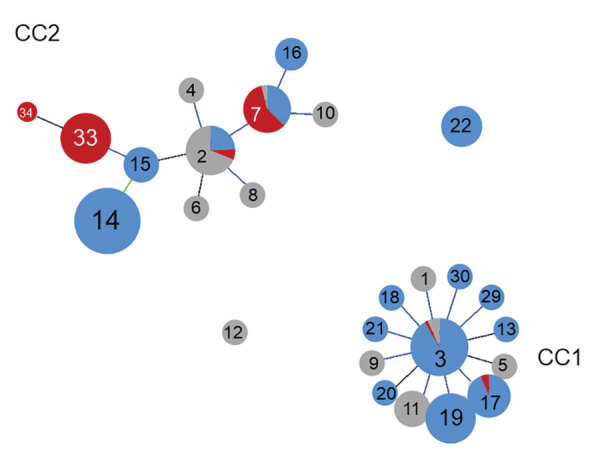
Relationships between CCs and STs for *Mycoplasma pneumoniae* isolates determined by goeBURST (http://www.phyloviz.net), Japan. Data were obtained from isolates including 470 strains from Japan during 2002–2019 and 62 strains isolated in the United Kingdom, the United States, China, and France. Blue circles indicate isolates from Japan during 2002–2016; red circles indicate isolates from Japan during 2018–2019; and gray circles indicate isolates from the United Kingdom during 1967–2010, the United States during 1944–1994, China during 2014, and France during 1981. Among all isolates, 26 STs were identified. CC, clonal complex; ST, sequence type.

We determined relationships between CC and STs according to goeBURST (Figure 2). The data include 470 strains from our study and 62 strains registered in the MLST database from other countries. Of the 2 CC clusters, the CC1 centered on ST3, and CC2 centered on ST2. ST7 and ST33, which were prevalent during 2018–2019, belonged to CC2. Although ST14 was the most prevalent member of CC2 until 2016, ST33 replaced it and increased during 2018. Both ST14 and ST33 were derived from ST15 and showed a single-locus variant of the *adk* gene. We registered ST34, which was derived from ST33, as a new ST.

Results for P1 typing of *M. pneumoniae* showed that STs belonging to CC1 were type 1 and STs belong to CC2 were type 2. ST14, ST15, ST33, and ST34 belonged to type 2a, a subtype of P1 type 2.

## Conclusions

In Japan, prevalence of macrolide-resistant *M. pneumoniae* has decreased recently and rapidly. Other study groups have reported similar trends (*8*). However, in countries in Asia other than Japan, the resistance rate has remained high in China (*3*) and South Korea (*1*). In the European Union, the overall rate is low, but has varied by country. Macrolide-resistant *M. pneumoniae* was not detected in Sweden during 1996–2013 (*9*), and the rate has been consistently low in Germany (1.9%–3.6%) (*10*). Because of tight control of antimicrobial drug prescriptions, Sweden shows extremely low use of macrolides (*11*) compared with for more frequent use in countries in Asia (*12*), where excessive use of macrolides is likely to affect selection and increase of drug-resistant strains.

In Japan, macrolide consumption has decreased gradually after the 2011–2012 outbreak of macrolide-resistant *M. pneumoniae* infections (http://amrcrc.ncgm.go.jp/surveillance/index.html), which may have contributed to the decrease in drug-resistant strains. In addition, the outbreak was followed by approval of tosufloxacin, a quinolone agent, for children with macrolide-resistant *M. pneumoniae* infection who fail to respond clinically to macrolides within 3 days. Approval of tosufloxacin as a treatment for these *M. pneumoniae* infections might have also contributed to the decrease in the macrolide resistance rate.

Our MLST results showed that predominant STs during 2018–2019 differed from those during 2016. *M. pneumoniae* could not be collected for our survey during 2017. This factor was a serious limitation because changes in STs might have occurred at that time. During 2018, strains identified as ST7 and ST33 were almost all susceptible and represented a major difference from previous findings. ST33, which contained a single-locus variant of the *adk* gene in ST15, was identified in Japan during 2018 and displaced ST14, the major ST among susceptible strains isolated during 2015–2016. The cause of ST replacement might have been acquisition of specific antibodies against epidemic *M. pneumoniae* in children and teenagers, which gave rise to a single-locus variant in its place.

CC1 is a circular clonal complex that spread from ST3 at the center toward different STs showing a single-locus variant. CC2 has extended from its original center at ST2 to include other STs with 1 or more mutated allele. STs belonging to CC2 seen to be more diverse than STs of CC1. Diversification has also been observed in type 2 of the P1 gene, corresponding to CC2 (*13*).

In conclusion, STs in *M. pneumoniae* isolates differed by area and year in Japan (*1*,*7*,*14*). Thus, MLST analysis is helpful in understanding worldwide trends among pathogenic *M. pneumoniae*.
